# Supplementary material for: Getting along to get ahead: The role of social context in tournament promotion and reward systems
Source: PLoS One. 2021 Sep 20;16(9):e0257389. doi: 10.1371/journal.pone.0257389 (PMC8452042; doi:10.1371/journal.pone.0257389)
Supplement: Attachment — Submitted filename: Rebuttal letter.docx [file pone.0257389.s002.docx]

**Editor Opening comment**

Dear Dr. Banks,

Thank you for submitting your manuscript to PLOS ONE. After careful consideration, we feel that it has merit but does not fully meet PLOS ONE’s publication criteria as it currently stands. Therefore, we invite you to submit a revised version of the manuscript that addresses the points raised during the review process.

Kind regards,

Zulqurnain Ali, PhD

Academic Editor

PLOS ONE

**Author response**

Thank you again for the opportunity to revise our submitted work.

**We look forward to receiving your revised manuscript.**

**Additional Editor Comments (if provided):**

**Please read respond to the following reviewers comments:**

This article is interesting and its quality is good. I have few concerns

**Reviewer comment**

1. The article is long, I notice few repetitions of the same ideas. Authors should try to be concise and avoid unnecessary repetitions without affecting the quality of the contents.

**Author response**

We streamlined the original submission by 2 pages with an emphasis on removing any redundancy. If there is a particular area where you are concerned about redundancy please let us know.

**Reviewer comment**

2. The references are quite old. The current state of the research is not apparent in the article. Authors should cite latest articles in the field

**Author response**

We have added 5 references to current work since our original submission that includes articles which are from 2019, 2020, or in press.

Arend, R. J. 2019. Cheat to win: How to hack tournament theory. *Business Research Quarterly, 22*(4): 216-225.

Ekinci, E., Kauhanen, A., & Waldman, M. 2019. Bonuses and promotion tournaments: Theory and evidence. *The Economic Journal, 129*(622): 2342-2389.

Guiteierrez, C., Obloj, T., & Frank, D. H. (2020). Better to have led and lost than never to have led at all? Lost leadership and effort provision in dynamic tournaments. *Strategic Management Journal, 42*, 774-801.

Li, J., Shi, W., Connelly, B., Yi, X., & Qin, X. (in press). CEO Awards and Financial Misconduct. *Journal of Management*.

Pruijssers, J. L., Heugens, P. P., & Van Oosterhout, J. (2020). Winning at a losing game? Side-effects of perceived tournament promotion incentives in audit firms. *Journal of Business Ethics, 162*(1), 149-167.

**Reviewer comment**

3. The authors mix two citation formats. Please follow the journal citation style.

**Author response**

We have checked our citation format against the style guide for PLOS One to ensure consistency in the journal citation style.

**Reviewer comment**

4. Proofread your paper from an English copy editor to improve the quality of language across the manuscript.

**Author response**

We have performed an additional review of our manuscript for grammar and quality of language.
